# Cross-Reaction, Enhancement, and Neutralization Activity of Dengue Virus Antibodies against Zika Virus: A Study in the Mexican Population

**DOI:** 10.1155/2019/7239347

**Published:** 2019-09-02

**Authors:** Mayra R. Montecillo-Aguado, Alfredo E. Montes-Gómez, Julio García-Cordero, Josselin Corzo-Gómez, Héctor Vivanco-Cid, Gabriela Mellado-Sánchez, J. Esteban Muñoz-Medina, Benito Gutiérrez-Castañeda, Leopoldo Santos-Argumedo, César González-Bonilla, Leticia Cedillo-Barrón

**Affiliations:** ^1^Departamento de Biomedicina Molecular, CINVESTAV IPN, Av. IPN # 2508 Col. San Pedro Zacatenco, Ciudad de México, Mexico; ^2^Laboratorio Multidisciplinario en Ciencias Biomédicas, Instituto de Investigaciones Médico-Biológicas, Universidad Veracruzana, Veracruz, Mexico; ^3^Unidad de Desarrollo e Investigación en Bioprocesos, Escuela Nacional de Ciencias Biológicas, Instituto Politécnico Nacional, Ciudad de México, Mexico; ^4^Laboratorio Central de Epidemiología, Centro Médico Nacional La Raza, Instituto Mexicano del Seguro Social, Ciudad de México, Mexico; ^5^Departamento de Inmunología, UMF Facultad de Estudios Superiores-Iztacala, Universidad Nacional Autónoma de México, Av. de los Barrios 1, Los Reyes Iztacala, Edo. de México, Tlalnepantla, Mexico; ^6^Laboratorio Central de Epidemiología, Coordinación de Vigilancia Epidemiológica, Instituto Mexicano del Seguro Social, Ciudad de México, Mexico

## Abstract

Zika virus (ZIKV), an emerging mosquito-borne flavivirus, has quickly spread in many regions around the world where dengue virus (DENV) is endemic. This represents a major health concern, given the high homology between these two viruses, which can result in cross-reactivity. The aim of this study was to determine the cross-reacting antibody response of the IgM and IgG classes against the recombinant envelope protein of ZIKV (rE-ZIKV) in sera from patients with acute-phase infection of different clinical forms of dengue, i.e., dengue fever (DF) and dengue hemorrhagic fever (DHF) (before the arrival of ZIKV in Mexico 2010), as well as acute-phase sera of ZIKV patients, together with the implications in neutralization and antibody-dependent enhancement. Differences in IgM responses were observed in a number of DF and DHF patients whose sera cross-reacted with the rE-ZIK antigen, with 42% recognition between acute-phase DHF and ZIKV but 27% recognition between DF and ZIKV. Regarding IgG antibodies, 71.5% from the DF group showed cross-reactivity to rE-ZIKV in contrast with 50% and only 25% of DHF and ZIKV serum samples, respectively, which specifically recognized the homologous antigen. The DHF group showed more enhancement of ZIKV infection of FCR*γ*-expressing cells compared to the DF group. Furthermore, the DHF group also showed a higher cross-neutralizing ability than that of DF. This is the first report where DF and DHF serum samples were evaluated for cross-reactivity against Zika protein and ZIKV. Furthermore, DENV serum samples cross-protect against ZIKV through neutralizing antibodies but at the same time mediate antibody-dependent enhancement in the sequential ZIKV infection.

## 1. Introduction

Zika virus (ZIKV) is a mosquito-borne member of the Flaviviridae family of single-stranded positive-sense RNA viruses. This virus is transmitted mainly by female *Aedes aegypti* mosquitoes, besides other mosquito species [1]. ZIKV remained a virus of low importance for many years, until its emergence, when it caused an epidemic outbreak after its introduction into Brazil in 2014 [2, 3]. After this, ZIKV rapidly spread across the Americas by 2016. Mexico has become a highly endemic area for arboviruses such as dengue virus (DENV), Chikungunya virus, and ZIKV, with the former two appearing in Mexico in 2015. In February 2016, the World Health Organization declared the spread of ZIKV a Public Health Emergency of International Concern [4]. In the past, most ZIKV infections were asymptomatic or mild, with self-limiting acute febrile illness accompanied by arthralgia, conjunctivitis, and rash [5, 6]. However, the more recent outbreaks have been shown to cause severe neurological complications such as congenital microcephaly *in utero* or at birth and Guillain–Barré syndrome in adults [7, 8]. In addition, autoimmune complications and optical lesions have been reported [9], as well as severe malformations in fetuses and newborns [10–12], possibly occurring as a result of the tropism of ZIKV for neural progenitor cells and its effects in differentiation [13] and apoptosis triggered after infection [14].

The full-length genome of ZIKV encodes three structural proteins, namely, capsid (C), precursor membrane (prM), and envelope (E), as well as seven nonstructural proteins (NS1, NS2a, NS2b, NS3, NS4a, NS4b, and NS5) [15]. Similar to other flaviviruses, structural protein E is the most exposed molecule on the virus. This protein is considered a major antigenic target for antibody responses in dengue patients [16, 17]. It also mediates virus entry through the putative receptor-binding sites for host cells (not fully identified thus far). Protein E of ZIKV is arranged in three functional and structural domains (DI, DII, and DIII). Bioinformatics analyses have shown that the E proteins of DENV type 2 (DENV2) and ZIKV share 53.9% amino acid sequence identity [18, 19]. This finding is very important considering that DENV serotypes 1, 2, 3, and 4 have been widely circulating throughout Mexico for many years. Thus, several studies have addressed the role of humoral immunity in the cross-reactivity between DENV and ZIKV using antibodies and sera from infected patients [18, 20–22], and these studies collectively showed that *in vitro*, some DENV antibodies are highly cross-reactive to ZIKV and vice versa.

Antibody-dependent enhancement (ADE) is a major concern owing to the high structural similarity between ZIKV and DENV envelope proteins, with ZIKV being a recently introduced pathogen for which dengue immune targets have not developed protection yet. Monoclonal antibodies (mAbs) generated from plasmablasts from acute or convalescent ZIKV-infected patients have shown the ability to enhance DENV infection *in vitro*, and some studies have even found a positive correlation between cross-reactive levels and ADE [20]. Meanwhile, mAbs obtained from DENV-infected patients can also enhance ZIKV infection. These data suggest that immunity to DENV might drive ZIKV replication and have direct implications in the pathogenesis [23]. The development of preexisting immunological memory against DENV is a worrying phenomenon in ZIKV infections, and this issue has been evaluated from perspectives such as acute versus convalescent, number of infections (primary/secondary, etc.), and infectious serotype. Furthermore, many mAbs have been identified against cross-reactive or type-specific epitopes from samples from a small number of individuals. However, there is no information regarding the cross-reactivity of immune sera from patients with different clinical forms of dengue to ZIKV either enhancing or neutralizing infection [24].

Here, we evaluated the cross-reactivity and functionality of antibodies in samples against ZIKV of immune sera from acute-phase dengue fever (DF) and dengue hemorrhagic fever (DHF) patients from a Mexican population in the Veracruz state. The serum samples were diagnosed on the basis of RT-PCR, serology, and clinical characterization. The serum samples were obtained in 2010 before the first Zika fever case was reported in Mexico; therefore, these patients had not been previously exposed to ZIKV. In addition, serum samples from acute-phase ZIKV-infected patients were also used to determine their reactivity to recombinant E proteins of ZIKV and DENV2 (rE-ZIKV and rPrE-DENV2, respectively), together with the ADE and neutralization of ZIKV and DENV. Levels of cross-reactivity, cross-neutralization, and enhancing antibodies were analyzed in the serum samples of the DF and DHF groups.

## 2. Materials and Methods

### 2.1. Patient Samples

The study protocol was approved by the institutional review board of Veracruz University's Institute for Biomedical Research Ethics Committee (protocol number 18/2010). In this retrospective study, blood samples were collected from patients aged 6–60 years 5–7 days after the onset of symptoms, and RT-PCR diagnosis was performed (at that time, ZIKV had not been reported in Mexico [25]). The positive patients were classified as DF or DHF based on clinical and laboratory criteria according to World Health Organization criteria [26]. All DHF patients demonstrated thrombocytopenia (platelet count < 100, 000/mm^3^) and positive findings in the tourniquet test; meanwhile, DF patients showed mild febrile disease. The ZIKV serum samples used in this study were collected at Veracruz University's Institute for Biomedical Research and the National Medical Center “La Raza” in Mexico City in 2016. Patients aged 4–68 years of age were diagnosed with ZIKV infection by RT-PCR and ELISA (IBL International Corp., Hamburg, Germany) 1–12 days after the onset of symptoms. As negative control subjects, we studied 150 healthy individuals aged 3–98 years from Fresnillo, Zacatecas, an area with very low endemicity for DENV/ZIKV in Mexico.

### 2.2. Viruses and Cells

The DENV2 New Guinea reference strain and ZIKV (KU922960.1) were propagated in C3/36 cells derived from *Aedes albopictus*. These cells were grown in minimum essential medium (pH 7.2) supplemented with 10% fetal bovine serum (FBS), 1 mM sodium pyruvate, 2 mM l-glutamine, and 1X nonessential amino acids (NEAA) (all from FBS; Gibco, Carlsbad, CA, USA) and incubated at 34°C. DENV2 and ZIKV stocks were prepared by infecting a monolayer of C6/36 cells with 80–85% confluence for 24–48 h until a cytopathic effect was observed. The supernatant was collected, cleared of cells by centrifugation at 1,000 *×g* for 10 min at 4°C, and concentrated in 0.22 *μ*m Amicon columns to 1/10 of the starting volume. Next, 1/10 volume of sucrose-phosphate-glutamate stabilizer (2.18 mM sucrose, 38 mM monobasic K_2_HPO_4_, 72 mM dibasic K_2_HPO_4_, and 60 mM l-glutamic acid) was added, and the stocks were aliquoted and stored at −80°C until further use.

Vero and K562 (CCL-81 and CCL-243, respectively; ATCC) cell lines were cultured in RPMI medium (pH 7.8; Gibco) supplemented with 5% FBS, 2 mM l-glutamine, 1X NEAA, and 1X vitamins and incubated in a CO_2_ atmosphere at 37°C.

*Drosophila melanogaster* Schneider 2 (S2) cells (Gibco) were grown in *Drosophila* Schneider 2 culture medium supplemented with 10% FBS and 2 mM l-glutamine and incubated at 28°C in the absence of CO_2_.

### 2.3. Vector Construction

The full-length ZIKV envelope sequence was synthesized (Gene Scr Biotech Corp., New Jersey, USA) based on the cDNA sequence, including nucleotides from 918 to 2,429 bp (1,512 bp) (GenBank KU922960.1), flanked by *Kpn*I and *Xba*I, into the pJET1.2 transfer vector. Therefore, the plasmid pJET1.2/EZIKV was digested with the restriction enzymes *Kpn*I and *Xba*I (New England Biolabs), and the obtained sequence was purified and subcloned into the pMT-BiP/V5-His expression plasmid of *Drosophila melanogaster* (Invitrogen, Carlsbad, CA, USA), which was also previously digested with the same enzymes. Either the E sequence encoding fragment, the linearized plasmid, or the resultant plasmid pMT/E-ZIKV was purified with a QIAquick Gel Extraction kit (Qiagen) following the manufacturer's instructions.

### 2.4. Transient Expression of the Recombinant Proteins

The plasmid pMT/E-ZIKV was transiently and stably transfected into *Drosophila* S2 cells (supplemented with 10% FBS) as described elsewhere. After induction, S2 cells were subjected to immunofluorescence analysis as described previously [27]. Briefly, S2 cell monolayers were washed with phosphate-buffered saline (PBS) (Triton), fixed/permeabilized using 2% paraformaldehyde, blocked with 10% goat serum, and stained for 60 min with a mAb against E-DENV protein [28].

### 2.5. Stable Transfectant Cells

Both the plasmids pMT/E-ZIKV (described above) and pMTPrME/DV2 (kindly donated by Dr. Philippe Despres, Institut Pasteur) were used to stably cotransfect *Drosophila* S2 cells with a plasmid containing the blasticidin resistance gene (pCoBlast; Invitrogen USA) using calcium phosphate according to the manufacturer's instructions (Invitrogen, USA). The S2 cells were incubated at 28°C, and blasticidin selection was performed at a final concentration of 25 *μ*g/mL every 4 days over 3 weeks until stable colonies appeared. Once stable transfectants appeared, 500 mM CuSO_4_ was used to induce the expression of the recombinant protein. Protein expression was evaluated daily for 16 days post induction. The protein concentration was quantified using the Bradford method and evaluated by western blot analysis. The secreted recombinant proteins were concentrated on 10 kDa columns (Millipore, Massachusetts, USA) to a final volume of 10 mL [27].

### 2.6. Preparation and Characterization of Recombinant Proteins

The supernatants of stable pMT/E-ZIKV-transfected S2 cells were collected and centrifuged at 1,000 *×g* for 20 min. Then, the supernatant was concentrated in 10 kDa and 30 kDa columns. Next, the expression of the rE-ZIKV protein was evaluated using western blot analysis. For rE-ZIKV, the protein (30 *μ*g per lane) was prepared with a loading buffer containing 2-mercaptoethanol and boiled for 10 min at 95°C. Then, the supernatants were electrophoresed by sodium dodecyl sulfate-polyacrylamide gel electrophoresis (SDS/PAGE) on a 10% (wt/vol) polyacrylamide gel and transferred to a nitrocellulose membrane. Blots were blocked for 1 h in 5% (wt/vol) milk in PBS with 0.1% Tween, and then, the membrane was incubated with mAbs against E-DENV protein for 1 h [28]. The blots were washed and incubated with HRP-conjugated goat anti-mouse secondary antibody (1030–05, Southern Biotech; Birmingham, USA) for 1 h. The blots were developed using SuperSignal West Femto Maximum Sensitivity Substrate (Thermo Fisher Scientific, USA) on a Bio-Rad Molecular Imager ChemiDoc.

### 2.7. ELISA for Cross-Reactivity Evaluation

For ELISA, Nunc MaxiSorp plates (M2936; Sigma, USA) were coated overnight at 4°C with rE-ZIKV or rPrME-DENV2 protein diluted in carbonate buffer (3 *μ*g/mL, pH 9.4). Plates were washed with PBS containing 0.1% Tween and blocked with PBS containing 5% (wt/vol) bovine serum albumin (BSA) for 1 h. Next, serum was serially diluted in PBS with 5% (wt/vol) BSA and added to the plates for 1 h. Peroxidase-conjugated anti-human IgM and IgG antibodies (627520, Invitrogen; 97225, Abcam) were added for 1 h before developing the plates using an *o*-phenylenediamine substrate (P9029; Sigma, USA) as the chromogen and H_2_O_2_ as the substrate's enzyme in citrate buffer (pH 5.6).

### 2.8. Antibody-Dependent Enhancement Assay

Sera from the patients and control individuals were serially diluted in 50 *μ*L RPMI containing 2% (vol/vol) FBS, antibiotics, and l-glutamine. Next, ZIKV or DENV2 at 2.5 multiplicities of infection (MOI) in a volume of 50 *μ*L was added to each serum dilution and incubated for 2 h at 37°C. The serum-virus mix was used to infect 5 × 10^4^ K562 cells for 24 h at 37°C. Flow cytometric staining for both the neutralization and ADE assays was performed with the fixing/permeabilizing solution Cytofix/Cytoperm (554714; BD, USA), followed by washing with 1X Perm/Wash Buffer according to the manufacturer's protocol. Cells were stained using the pan-flavivirus 4G2 antibody for 30 min, followed by the anti-mouse IgG Alexa-Fluor 488 antibody (A11029; Life Technologies, USA) for 30 min. The frequency of infected cells was determined using flow cytometry and was defined as the percentage of 4G2-positive stained cells [29].

### 2.9. Viral Neutralization Assay

Sera from the patients and control individuals were serially diluted in 50 *μ*L RPMI containing 2% (vol/vol) FBS, antibiotics, and 2 mM l-glutamine. Then, 2.5 MOI of ZIKV or DENV2 in a volume of 50 *μ*L was added to each serum dilution and incubated for 1 h at 37°C. This serum-virus mix was used to infect 2.5 × 10^4^ Vero cells in 96-well plates in a final volume of 100 *μ*L for 2 h. The complexes were then removed and washed with 1X PBS to further replenish with fresh supplemented culture media and incubated for 24 h at 37°C. Next, the cells were stained for flow cytometric analysis as described above.

### 2.10. Statistical Analysis

Statistical comparisons were made with Student's *t*-test for the determination of the differences between two sample means. For more than two samples, nonparametric ANOVA was used; *P* ≤ 0.05 was considered significant, and cutoff points were established by adding two standard deviations to the mean of healthy individuals from nonendemic areas. Prism v.6 software was used for statistical analyses (GraphPad Inc., La Jolla, CA, USA).

## 3. Results

### 3.1. Cloning of the E-ZIKV Recombinant Sequence

The structural similarity among the E proteins of different flaviviruses, including DENV (Tonga/74) and ZIKV (PRVABC59), has been reported [18, 30]. In the present study, we used Mexican strains to enable the comparison of protein E identity among DENV2 [31] and ZIKV isolated in Mexico [32] to address the homology between the two viral strains circulating in Mexico. E protein sequences of ZIKV and the four DENV serotypes were analyzed, as shown in Table 1. However, only serotype 2 was evaluated, because it is the most prevalent in Mexico among the four serotypes. Furthermore, the whole E proteins of the ZIKV and DENV strains used in this study showed high sequence identity of around 58.57% (Table 1).

**Figure 1:**
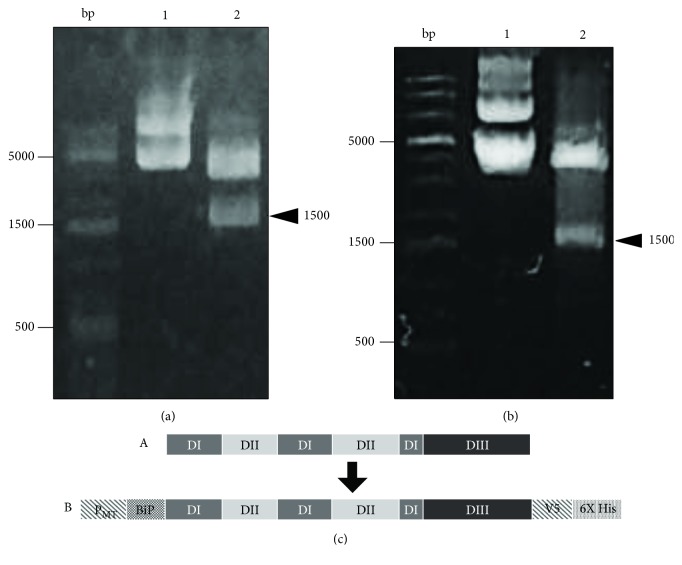
Cloning of the E-ZIKV gene of ZIKV. (a) Digestion of the plasmid pJET2.1 with restriction enzymes *Xba*I and *Kpn*I. Lane 1: undigested plasmid pJET2.1; lane 2: digested pJET2.1 plasmid yielding a 1,500 bp fragment. (b) Digestion of the plasmid pMTBiP/V5-His B with the restriction enzymes *Xba*I and *Kpn*I. Lane 2: restriction analysis of the construct pMT/E-ZIKV, which yields a 1,500 bp fragment. (c) A: diagram of the full-length E-ZIKV sequence; B: a schematic representation of rE-ZIKV flanked by the elements of the plasmid pMT, BiP, V5, and 6X His.

The digestion of the pJET2.1 plasmid containing the E protein sequence with the restriction enzymes *Xba*I and *Kpn*I yielded a 1,500 bp insert, as expected (Figure 1(a)). This sequence was subcloned in a pMT-BiP/V5-His plasmid, and restriction analysis of the pMTE-ZIKV construct was performed with the same enzymes, which yielded the corresponding 1,500 bp band (Figure 1(b)). The construct pMTE-ZV is illustrated in Figure 1(c).

### 3.2. Expression and Purification of rE-ZIKV in S2 Cells

**Figure 2:**
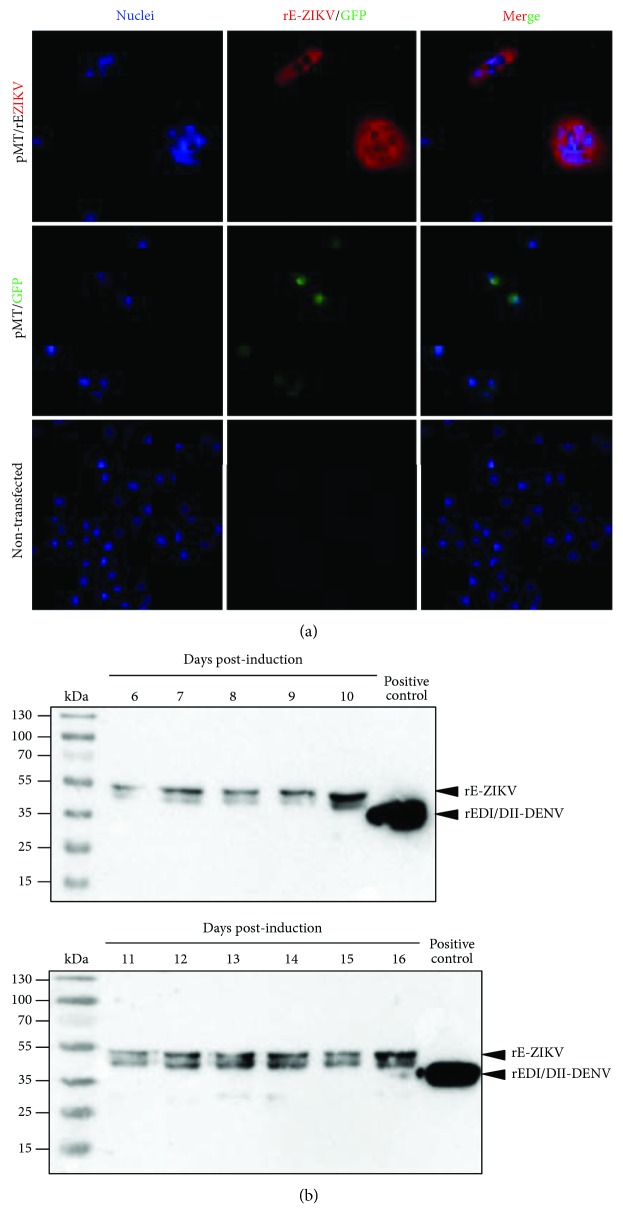
Expression and purification of rE-ZIKV in S2 cells. (a) Cytoplasmic expression of rE-ZIKV in *Drosophila melanogaster* S2 cells after transfection with pMT/E-ZIKV and induction with CuSO_4_. The envelope protein was detected using a monoclonal antibody anti-E protein of DENV (C-21), which cross-reacts with E-ZIKV. As a positive control, S2 cells were transfected with a plasmid encoding the green fluorescent protein. (b) Secretion kinetics of the rE-ZIKV protein from day 6 to 16 after induction with CuSO_4_. The supernatants of the stably transfected S2 cells were harvested and analyzed using western blot analysis. The immunodetection was performed with anti-E protein (C-21).

The expression of the rE-ZIKV protein driven by the metallothionein promoter was assessed. From day 6 after induction with CuSO_4_, S2 cells were treated with brefeldin A and evaluated for intracellular protein expression by immunofluorescence (Figure 2(a)). Positive immunoreactivity was detected using a specific anti-E protein antibody [28]. This monoclonal anti-E antibody has been shown to bind to the whole virus in previous studies conducted by our group [28]. Furthermore, the supernatants were analyzed by western blotting using the same anti-E antibody. The observed bands were consistent with the predicted sizes (approximately 55 kDa; Figure 2(b)). Then, to produce a larger stock of the recombinant protein for subsequent assays, stably transfected cells were expanded, and the optimal time to harvest rE-ZIKV protein was established on day 10 after induction (Figure 2(b)).

### 3.3. Cross-Reactivity of Serum from Acute DENV Infections to rE-ZIKV Protein

A large number of studies have shown that E proteins of ZIKV and DENV have a high degree of structural similarity, also sharing many antigenic sequences. Considering that ZIKV is established within highly dengue-endemic areas, the possibility of preexisting dengue-induced antibodies that cross-react with ZIKV is high.

Thus, we investigated the magnitude of cross-reactivity of IgM and IgG antibody responses to rE-ZIKV protein from acute-phase serum samples with different clinical forms of DENV. The serum samples used in this study were collected on days 5–7 from the onset of fever. All the samples were collected in 2010, before the arrival of ZIKV in the country (2016). We chose Mexican individuals who had RT-PCR-confirmed symptomatic DENV infections, i.e., symptomatic DF (*n* = 150) and DHF (*n* = 45); samples from ZIKV-infected patients from outbreak 2016 (*n* = 88); and uninfected individuals from a nonendemic area (*n* = 150) (Table 2).

**Figure 3:**
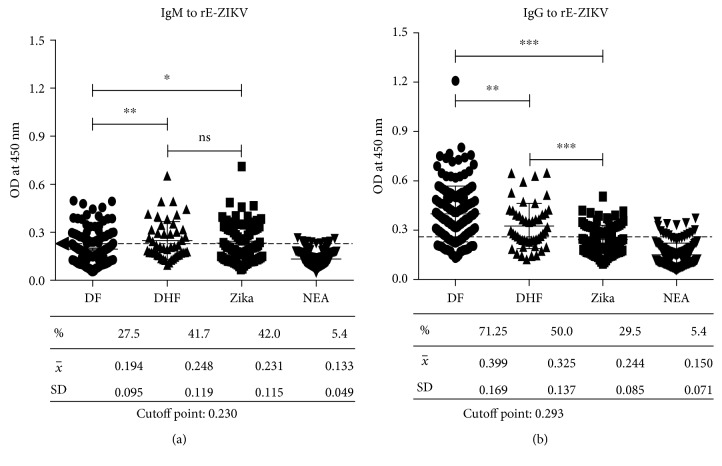
Cross-reaction of acute DF and DHF serum samples against rE-ZIKV. ELISA plates were coated with 3 *μ*g/mL of rE-ZIKV, and serial dilutions of each sample were used. The graph shows all the serum samples at 1 : 800 dilutions. Horseradish peroxidase-bound goat anti-human IgG or IgM was used as a secondary antibody. (a) Type-specific IgM antibody levels binding to rE-ZIKV. (b) Type-specific IgG antibody levels binding to rE-ZIKV. Bars represent the mean ± SD. ^∗^*P* < 0.05, ^∗∗^*P* < 0.01, and ^∗∗∗^*P* < 0.001.

We analyzed the IgM and IgG cross-reactivity of all the samples described. Sera from different groups were tested at different dilutions in ELISA plates coated with 3 *μ*g/mL of the rE-ZIKV antigen. The cutoff OD value for a positive result was defined as two standard deviations from the mean OD of sera from uninfected individuals from a nonendemic area (NEA). The IgM cross-reactive responses were analyzed from the two clinical forms DF and DHF; the samples showed wide cross-reactivity (1 : 800) against rE-ZIKV. The total of DHF and DF samples included in the groups showed cross-reactivities of 41.7% and 27.5%, respectively, with the rE-ZIKV antigen. Interestingly only 42% of ZIKV-positive samples recognized the homologous protein rE-ZIKV. Only two of the negative sera reacted to the rE-ZIKV protein. This suggests that the specific IgM class antibodies present in the sera of patients with dengue cross-recognize the rE-ZIKV. Subsequently, IgG class cross-reactive antibodies against rE-ZIKV in DF and DHF patients were analyzed. Most of the samples from the NEA were negative against rE-ZIKV (95%). Among the different groups, we again observed a wide distribution of OD values in samples of both DF and DHF groups; however, interestingly, 71.3% of DF serum samples cross-reacted with rE-ZIKV protein, in contrast with 50% of the DHF serum samples. Finally, only 29.5% of the samples from patients with a Zika diagnosis recognized the rE-ZIKV protein. Nevertheless, a lower percentage of positive responders against rE-ZIKV were observed in patients with acute ZIV infection; thus, the memory response might have been due to the previous contact with DENV. These data reflect the similarities and abundances of common epitopes between DENV and ZIKV (Figure 3).

**Figure 4:**
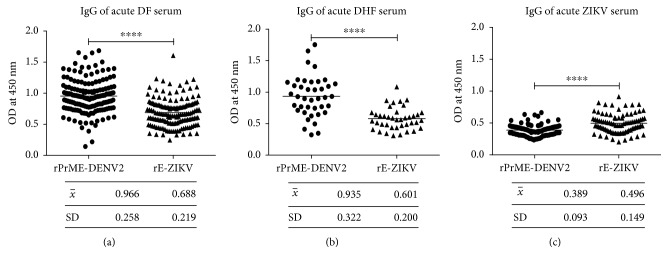
Comparative analysis of the cross-reactivity response against rPrME-DENV2 and rE-ZIKV from acute DF, DHF, and ZIKV serum samples. (a) Serum IgG cross-reactivity from patients with DF sera to rPrME-DENV2 and rE-ZIKV; (b) serum IgG cross-reactivity from patients with acute DHF sera to rPrME-DENV2 and rE-ZIKV; (c) serum IgG cross-reactivity of ZIKV sera to rPrME-DENV2 and rE-ZIKV. Bars represent the mean ± SD. ^∗^*P* < 0.05, ^∗∗^*P* < 0.01, and ^∗∗∗^*P* < 0.001.

When we compared the IgG antibody response at the same dilution (1 : 100) of the sera from patients with different clinical forms of dengue (DF and DHF) and Zika (all in the acute phase) against both recombinant antigens rE-ZIKV and rPrME-DENV2, the serum samples from DF and DHF patients against the two antigens showed significantly higher responses against the homologous rPrME-DENV antigen than against the antigen rE-ZIKV. We did not find any difference in the magnitude of the response between the two clinical forms DF and DHF. In contrast, Zika sera showed a lower reactivity against both antigens (Figure 4) but higher reactivity against the homologous antigen rE-ZIKV.

### 3.4. Serum Samples from DF and DHF Patients Possess an Enhancing Effect on ZIKV

In DENV-endemic areas, all four DENV serotypes frequently circulate at the same time or are cyclically replaced depending on the year. Thus, the highly cross-reactive antibodies observed above may have shown Fc*γ*-mediated enhanced uptake of the antibody-virus complex (the ADE phenomenon).

**Figure 5:**
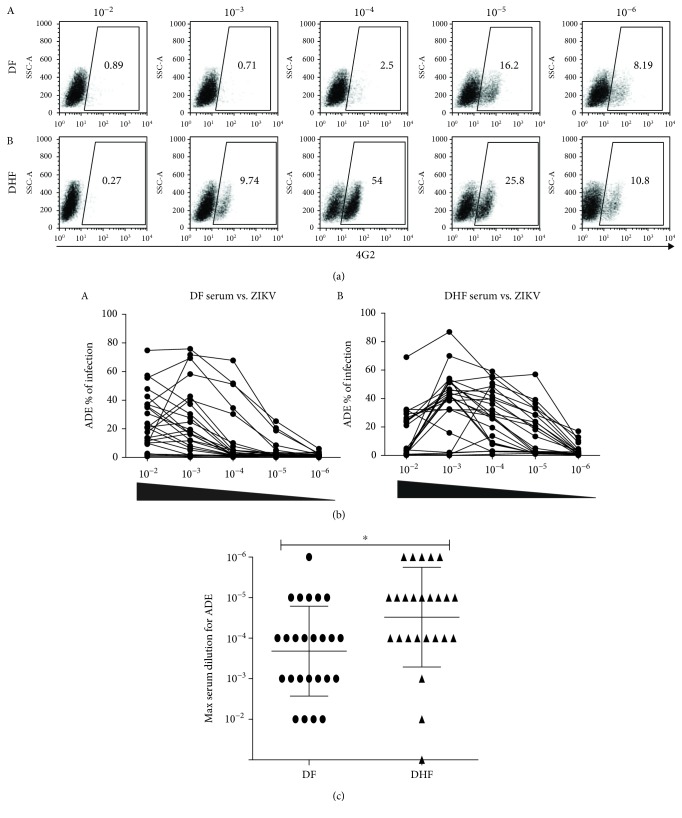
Antibody-dependent enhancement of ZIKV by DF and DHF sera. Serially diluted sera were mixed and incubated for 1 h with 1.25 × 10^5^ PFU of ZIKV and used to infect 5 × 10^4^ K562 cells for 24 h. Infection was analyzed by flow cytometry. (a) Representative dot plots of the ADE of one DF serum sample (A) and one DHF serum sample (B). (b) Enhancement of ZIKV by DF (A) and DHF (B) serum samples represented as percent of infection. (c) Comparison of both groups DF and DHF; each serum sample was graphed at the maximum dilution where at least 5% ADE was still observed. As a positive control, 4G2 serum was used for ADE. Bars represent the mean ± SD. ^∗^*P* < 0.05, ^∗∗^*P* < 0.01, and ^∗∗∗^*P* < 0.001.

Twenty-five serum samples randomly selected from each group were analyzed; DF and DHF (acute phase) groups were used to determine whether immunity against DENV had any effect in enhancing ZIKV infection *in vitro*. Serially diluted serum samples were incubated with ZIKV, and these mixtures were used to infect semiadherent K562 cells bearing the Fc-gamma receptor (FcR) for 24 h. The percentage of infection was determined using flow cytometry.  Figure 5(a) shows a representative dot plot for one serum sample of each clinical form: DF and DHF.  Figure 5(b) represents the whole group analyzed for both DF (A) and DHF (B); both groups showed broad diversity in the enhancement of ZIKV at lower dilutions. Meanwhile, few DF sera samples very efficiently enhanced ZIKV infection, which could reach up to 70% infection, with most of them decreasing this effect at higher dilutions. In contrast, in the DHF group, the enhancement of the infection was maintained even at dilutions as high as 10^−6^ (Supplementary Fig. [Supplementary-material supplementary-material-1]). Thus, when the two groups were compared, DHF sera showed a significantly stronger enhancement effect over ZIKV compared to DF sera.  Figure 5(c) shows a graph of both DF and DHF showing the higher dilution at which enhancement was still observed, considering 5% infection a positive result.

### 3.5. DENV-Induced Antibodies Cross-Neutralize ZIKV

**Figure 6:**
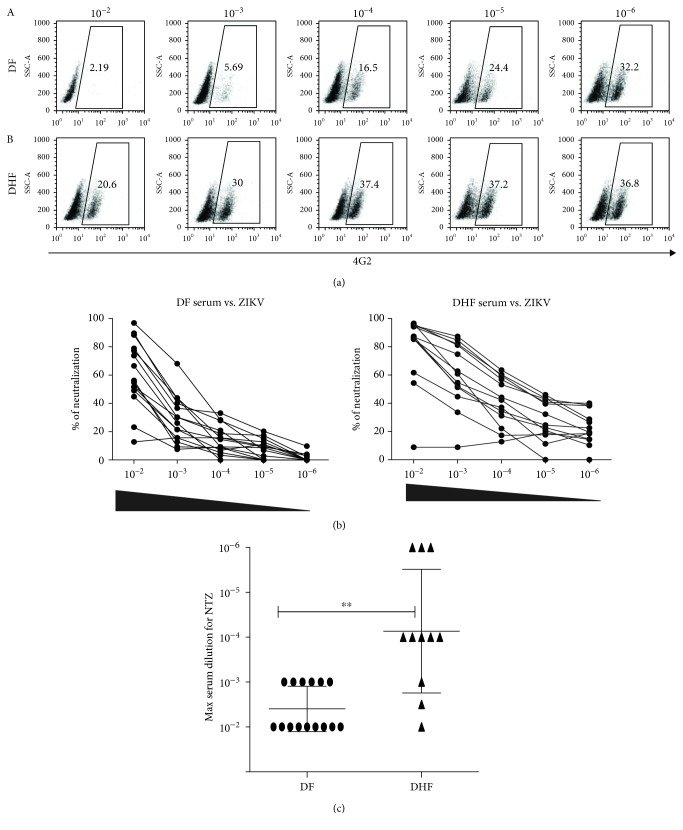
Neutralization of ZIKV by DENV sera. Serially diluted sera from DF and DHF patients were mixed with 7.5 × 10^5^ PFU of ZIKV and incubated for 1 h; these complexes were used to infect 2.5 × 10^5^ Vero cells for 24 h, and infection was evaluated by flow cytometry. (a) Representative dot plots of one DF serum sample (A) and one DHF serum sample (B). (b) Percent of neutralization of ZIKV with DF serum samples and DHF serum samples. (c) Comparison of both group DF and DHF sera; each sample was graphed at the maximum dilution where neutralization still was observed. As a positive control, 4G2 serum was used for ADE.

**Figure 7:**
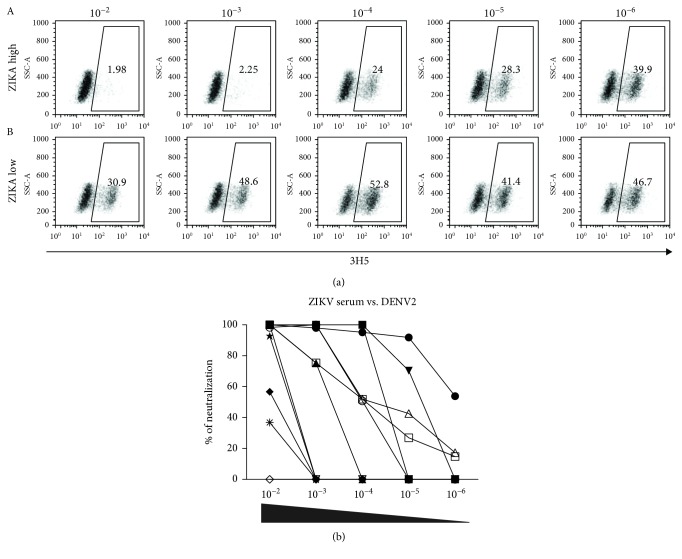
Neutralization of DENV by serum samples from acute-phase ZIKV-infected serum samples. Serially diluted sera from ZIKV-infected patients were mixed with 7.5 × 10^5^ PFU of DENV2 and incubated for 1 h; these complexes were used to infect 2.5 × 10^5^ Vero cells for 24 h, and infection was evaluated by flow cytometry. (a) Representative dot plots of two ZIKV serum samples, with high (A) and low (B) neutralizing activities against DENV2. (b) Percent of neutralization of DENV2 by sera from Zika-infected patients. Neutralization was based on Vero cells infected with DENV in the absence of immune sera, considering this as the highest infection rate reached (data not shown).

DENV infection with one dengue serotype will induce lifelong homologous protection, but the extent of cross-protection against other serotypes is still debated.  Table 1 shows high sequence identity and structural homology among E-DENV proteins. It is well known that the serotype-specific antibodies developed during DENV infection are mainly against domain III of the E protein (EDIII) [33]. However, as shown by our group and others, most of the memory IgG antibodies are cross-reactive antibodies that bind to domains I/II of the E protein (EDI/DII) [18, 34]. Whether these highly cross-reactive antibodies might also participate in protection against flaviviruses remains to be elucidated. To assess if the neutralizing activity of the serum samples with acute DF and DHF cross-neutralize ZIKV, we prepared serial dilutions from serum samples of DF and DHF mixed with ZIKV, and these mixtures were used to infect Vero cells for 24 h. Then, the percentage of neutralization was assessed using flow cytometry.  Figure 6(a) shows a representative cross-neutralizing assay with dot plots of the neutralization of ZIKV by DF and DHF serum samples, where strong cross-neutralization of ZIKV by DHF serum and lower cross-neutralization by DF serum can be observed. As shown in Figure 6(b), both groups showed strong neutralizing activities at higher concentrations. However, this phenomenon was lost in the DF group as the sera were diluted, while in the DHF group, neutralization remained. This effect is better observed in Supplementary Fig. [Supplementary-material supplementary-material-1], showing all dilutions used for both groups. Furthermore, Figure 6(c) shows the comparative graph of DF and DHF reported as the higher dilution at which the serum sample neutralizes between 40% and 60% viruses in the cytometry assay. We can conclude that DHF sera showed stronger cross-neutralization against ZIKV *in vitro*. The impact of these neutralizing antibodies on either protective immunity or disease severity after ZIKV infection remains to be elucidated. Interestingly, we found that the neutralizing curve did not show a sigmoidal form, perhaps due to the acute phase, where overactivation and different molecules, beside antibodies, may enhance the neutralizing effect. Finally, the cross-neutralizing activity of sera from ZIKV-infected patients against DENV2 was assessed using a flow cytometry-based assay. We observed different neutralization patterns in the samples analyzed; as shown in Figure 7(a), the dot plot represents two serum samples from ZIKV-infected patients, with strong and weak cross-neutralizing activities, respectively, against DENV.  Figure 7 represents the ZIKV-infected group overall and shows some samples with high neutralizing activity and others with very low neutralizing activity (Figure 7(b)). Although most of the acute ZIKV infection sera are highly likely to be immune to dengue, the capacity of an individual to neutralize DENV will depend on the memory cells triggered by ZIKV during the acute phase.

## 4. Discussion

When ZIKV was first discovered, it was considered a flavivirus of low importance. However, the virus started to assume dangerous proportions, with major outbreaks since 2007 in Asia and its subsequent spread in America. Several studies have demonstrated the high conservation among the antigenic and exposed protein E. Nevertheless, studies reveal that ZIKV is antigenically very similar to DENV [18, 19]. Thus, the arrival of ZIKV to highly endemic zones of DENV raises huge concerns in terms of the role of the preexisting immune response to DENV. Considerable data suggest the association between heterotypic infection and the risk of severe dengue disease, probably because antibodies against one serotype may enhance infections with heterologous serotypes (ADE) by promoting viral entry and infection through Fc receptor-expressing cells. Thus, the aim of this study was to evaluate the cross-reactivity levels of antibodies binding to rE-ZIKV in serum from acute-phase DF or DHF patients. This rE-ZIK antigen was expressed in *Drosophila*, and the cross-neutralizing and enhancing antibody infection was evaluated.

Although this is not the first report showing that preexisting anti-DENV antibodies are highly cross-reactive to ZIKV, to the best of our knowledge, this is the first study to show the influence of anti-DENV antibodies (differentiated for the clinical forms of dengue: DF or DHF) from endemic areas on ZIKV infection *in vitro*. We clearly demonstrated substantial cross-reactivity in serum samples from acute DENV and ZIKV against envelope proteins, although the varied responses of IgM among donors might be based on DENV exposure. In this case, a DENV-biased recall response might be responsible. Although interesting results and differences were found among the cross-reactivity against the rE-ZIKV protein, it is important to emphasize that many antibodies from natural infection do not bind to recombinant E proteins since they are directed to conformational epitopes present in the dimeric E protein [35].

Here, we used a denatured protein, and thus, some epitopes might have been absent. Nevertheless, E protein is a good target to evaluate the humoral response, given its immunogenicity. These factors explain why we did not find a tight correlation between the results using the recombinant protein and the assays where the whole viruses were used, such as the neutralizing and ADE assays. Some reports regarding such conformation-sensitive, dimer-dependent antibodies include the broadly neutralizing antibody that recognizes the envelope dimer epitope (EDE). This mAb was originally isolated from patients with dengue, and several mAbs binding the EDE1 epitope have been shown to potently neutralize ZIKV *in vitro* [36].

Many groups have documented that DENV immune serum may exacerbate disease through ADE, as a result of subneutralizing cross-reactive antibodies, and the same phenomenon might be observed through secondary infection with ZIKV. Therefore, we used acute-phase serum samples from the state of Veracruz, which is highly endemic for dengue in Mexico. Furthermore, the samples were taken in 2010, and according to epidemiological studies, ZIKV was not reported until 2015 [37]. We also used acute-phase ZIKV sera from a recent outbreak in 2016 in Veracruz. What we observed in the IgG response against the rEIII-ZIKV antigen in DF and DHF samples was clearly a memory response due to the DENV infections. Meanwhile, the Zika sera did not show a high cross-reaction against the rE-DV2 protein despite being from patients in an endemic area (Figure 4(c)).

In the current study, we also evaluated functional activity with the whole virus, such as neutralization of infection and the ADE phenomenon. In this regard, we found that DHF sera had a stronger enhancing effect on ZIKV infection in a K562 cell line model, where although serum from DF also enhanced infection at serum dilutions (1 : 100), it was quickly lost, compared to DHF, where 20% infection remained at the highest dilution. These data are in line with the long-known fact that ADE mainly explains the disease severity of DHF where higher viral loads are observed, both directly in different *in vitro* models and indirectly, where primary DENV-infected infants, whose mothers were immune to dengue, were found to be more susceptible to develop DHF [38, 39]. One of the studies also demonstrated that maternal antibodies against DENV decline at a constant rate and pass through three functional states: neutralization, enhancement, and antibody degradation [40].

Next, we assessed the neutralizing activity of DF and DHF sera against ZIKV. Several DENV infections with different serotypes will provide broad protection to all four serotypes [41]. Therefore, the cross-neutralizing activity of DENV immune sera was assessed against ZIKV. Surprisingly, DHF sera showed a stronger neutralizing activity compared to DF sera. The history of previous flaviviral infections must be considered, because a recent study showed that a DENV-naïve American traveler got infected by ZIKV, but *in vitro* neutralization assays showed that his serum could neutralize both DENV and ZIKV. It has been widely accepted that the neutralization of flaviviruses is a multihit event, in which an antibody concentration threshold must be reached to successfully neutralize the virus [42]. However, if this threshold is not reached, neutralization fails, and infection might be enhanced. An in-depth clinical study revealed a history of vaccination against Japanese encephalitis virus two years earlier, and thus, *in vitro* neutralization could be mediated by antibodies binding to common epitopes among flaviviruses [43]. The use of Mexican samples was valuable because this population is from highly endemic areas of DENV; furthermore, they might have been infected by more than one DENV serotype since more than one serotype could be circulating at the same time or cyclically. Thus, there is continuous exposure to DENV, which might drive the maintenance of ZIKV cross-neutralizing antibodies following DENV infection. Cross-neutralization can also be observed in DENV-infected travelers, in whom ZIKV cross-neutralizing antibodies are not maintained in the late convalescent phase. Continuous exposure to DENV is one of the factors explaining why the recent arrival of ZIKV to a highly DENV-endemic country such as Mexico differed from that in Brazil. In this respect, Pantoja et al. demonstrated that preexisting DENV antibodies fail to increase disease severity in macaques infected with ZIKV. They also analyzed B and T cell activation and cytokine profiles and concluded that DENV immune memory may modulate immune responses against ZIKV [44].

Moreover, high levels of cross-reactive antibodies have been found to be linked with a reduced probability of symptomatic DENV infection [45] and now of ZIKV infection as well. Despite a lesser number of samples from Zika patients' sera having been tested in the inverse system (Figure 7), it is remarkable that three of them still showed neutralizing activity at up to 10^−6^ dilution: two at 20% and one at 60% corroborating the behavior observed with DHF sera. However, some samples showed low neutralization capacity, which might be due to the primary response against ZIKV and previous encounters with other DENV serotypes but not DENV2. Recent studies using human mAbs generated from DENV-convalescent donors have shown both neutralizing and enhancing activities.

Although considerable data on cross-reactivity have been published recently, we emphasize that this is the first study where DF and DHF serum samples were examined. However, we found that DENV preimmune serum (from people with prior DENV exposure) can cross-protect through neutralizing antibodies, both those directed to domain III and those directed to domains I and II, which are more abundant, with low neutralizing ability but high occupancy. An interesting example is the fusion loop-specific antibody named 2A10G6, which neutralizes several flaviviruses *in vitro*, including ZIKV, DENV1–4, and yellow fever virus [46]. Meanwhile, the same bulk of antibodies may be implicated in pathology during sequential ZIKV infection through infection enhancement phenomena, which might exacerbate the disease in humans. Furthermore, a balance could arise with certain special characteristics or the immunological background of each individual. It should be noted that at the arrival of ZIKV in Mexico, a disastrous scenario was expected due to the immunological memory against dengue; however, epidemiological data show that in 2015 and 2016, there were no major cases of Zika in Mexico. Moreover, even in 2018, the number of cases was considerably low, with only four cases in 2019 so far. Thus, the immunity against the virus could be protective in some cases of ZIKV infection.

In conclusion, the current study demonstrates that DF patients in DENV-endemic areas present a greater IgG-type cross-reaction to the E-ZIKV protein and facilitate and neutralize the infection depending on the concentration of the antibodies. Meanwhile, patients with DHF maintain an ADE and more sustained neutralizing activity at higher serum dilutions. Further research is needed to identify the epitopes that these antibodies target.

## Figures and Tables

**Table 1:** Homology analysis between the E protein sequences of the four DENV serotypes and ZIKV.

	DENV1 (KM279419.1)	DENV2 (H171541.1)	DENV3 (HM171540.1)	DENV4 (HM171571.1)	ZIKV (KU922960.1)
DENV1 (KM279419.1)	100%	65.58%	69.37%	64.21%	57.44%
DENV2 (H171541.1)	—	100%	64.67%	66.34%	58.57%
DENV3 (HM171540.1)	—	—	100%	64.33%	61.05%
DENV4 (HM171571.1)	—	—	—	100%	62.56%
ZIKV (KU922960.1)	—	—	—	—	100%

**Table 2:** Characteristics of subjects analyzed.

Diagnosis	Stage of infection	Year of sample collection	*N*
Dengue fever (DF)	Acute	2010	150
Dengue hemorrhagic fever (DHF)	Acute	2010	45
Zika	Acute	2016	88
Nonendemic area (NEA)	—	2016	150

## Data Availability

All data used to support the finding presented here are available upon request to the corresponding author.

## Supplementary Materials

Supplementary Materials Supplementary Fig. 1: antibody-dependent enhancement of ZIKV by DF and DHF sera. Supplementary Fig. 2: neutralization of ZIKV by DENV sera at different dilutions of DHF sera.

## References

[1] Musso D. (2015). Zika virus transmission from French Polynesia to Brazil. *Emerging Infectious Diseases*.

[2] Faria N. R., Quick J., Claro I. M. (2017). Establishment and cryptic transmission of Zika virus in Brazil and the Americas. *Nature*.

[3] Zanluca C., Melo V. C. A., Mosimann A. L. P., Santos G. I. V., Santos C. N. D., Luz K. (2015). First report of autochthonous transmission of Zika virus in Brazil. *Memórias do Instituto Oswaldo Cruz*.

[4] http://www.who.int/news-room/detail/01-02-2016-who-statement-on-the-first-meeting-of-the-international-health-regulations-(2005)-(ihr-2005)-emergency-committee-on-zika-virus-and-observed-increase-in-neurological-disorders-and-neonatal-malformations

[5] Duffy M. R., Chen T. H., Hancock W. T. (2009). Zika virus outbreak on Yap Island, Federated States of Micronesia. *The New England Journal of Medicine*.

[6] Simpson D. I. (1964). Zika virus infection in man. *Transactions of the Royal Society of Tropical Medicine and Hygiene*.

[7] Cao-Lormeau V. M., Blake A., Mons S. (2016). Guillain-Barré Syndrome outbreak associated with Zika virus infection in French Polynesia: a case-control study. *The Lancet*.

[8] Oehler E., Watrin L., Larre P. (2014). Zika virus infection complicated by Guillain-Barré syndrome – case report, French Polynesia, December 2013. *Eurosurveillance*.

[9] Ventura C. V., Fernandez M. P., Gonzalez I. A. (2016). First travel-associated congenital Zika syndrome in the US: ocular and neurological findings in the absence of microcephaly. *Ophthalmic Surgery, Lasers & Imaging Retina*.

[10] Calvet G., Aguiar R. S., Melo A. S. O. (2016). Detection and sequencing of Zika virus from amniotic fluid of fetuses with microcephaly in Brazil: a case study. *The Lancet Infectious Diseases*.

[11] Mlakar J., Korva M., Tul N. (2016). Zika virus associated with microcephaly. *The New England Journal of Medicine*.

[12] Sarno M., Sacramento G. A., Khouri R. (2016). Zika virus infection and stillbirths: a case of hydrops fetalis, hydranencephaly and fetal demise. *PLoS Neglected Tropical Diseases*.

[13] Tang H., Hammack C., Ogden S. C. (2016). Zika virus infects human cortical neural progenitors and attenuates their growth. *Cell Stem Cell*.

[14] Dang J., Tiwari S. K., Lichinchi G. (2016). Zika virus depletes neural progenitors in human cerebral organoids through activation of the innate immune receptor TLR3. *Cell Stem Cell*.

[15] Lee I., Bos S., Li G. (2018). Probing molecular insights into Zika virus–host interactions. *Viruses*.

[16] Beltramello M., Williams K. L., Simmons C. P. (2010). The human immune response to Dengue virus is dominated by highly cross-reactive antibodies endowed with neutralizing and enhancing activity. *Cell Host & Microbe*.

[17] Xu M., Hadinoto V., Appanna R. (2012). Plasmablasts generated during repeated dengue infection are virus glycoprotein-specific and bind to multiple virus serotypes. *The Journal of Immunology*.

[18] Priyamvada L., Quicke K. M., Hudson W. H. (2016). Human antibody responses after dengue virus infection are highly cross-reactive to Zika virus. *Proceedings of the National Academy of Sciences of the United States of America*.

[19] Stettler K., Beltramello M., Espinosa D. A. (2016). Specificity, cross-reactivity, and function of antibodies elicited by Zika virus infection. *Science*.

[20] Bardina S. V., Bunduc P., Tripathi S. (2017). Enhancement of Zika virus pathogenesis by preexisting antiflavivirus immunity. *Science*.

[21] Collins M. H., McGowan E., Jadi R. (2017). Lack of durable cross-neutralizing antibodies against Zika virus from dengue virus infection. *Emerging Infectious Diseases*.

[22] Swanstrom J. A., Plante J. A., Plante K. S. (2016). Dengue virus envelope dimer epitope monoclonal antibodies isolated from dengue patients are protective against Zika virus. *MBio*.

[23] Keasey S. L., Pugh C. L., Jensen S. M. R. (2017). Antibody responses to Zika virus infections in environments of flavivirus endemicity. *Clinical and Vaccine Immunology*.

[24] Priyamvada L., Hudson W., Ahmed R., Wrammert J. (2017). Humoral cross-reactivity between Zika and dengue viruses: implications for protection and pathology. *Emerging Microbes & Infections*.

[25] Díaz-Quiñonez J. A., López-Martínez I., Torres-Longoria B. (2016). Evidence of the presence of the Zika virus in Mexico since early 2015. *Virus Genes*.

[26] World Health Organization (1997). *Dengue hemorrhagic fever: diagnosis, treatment, prevention and control*.

[27] Mellado-Sánchez G., García-Cordero J., Luria-Pérez R. (2005). DNA priming E and NS1 constructs–homologous proteins boosting immunization strategy to improve immune response against dengue in mice. *Viral Immunology*.

[28] León-Juárez M., García-Cordero J., Santos-Argumedo L. (2013). Generation and characterization of a monoclonal antibody that cross-reacts with the envelope protein from the four dengue virus serotypes. *APMIS*.

[29] Konishi E., Tabuchi Y., Yamanaka A. (2010). A simple assay system for infection-enhancing and -neutralizing antibodies to dengue type 2 virus using layers of semi-adherent K562 cells. *Journal of Virological Methods*.

[30] Sirohi D., Chen Z., Sun L. (2016). The 3.8 A resolution cryo-EM structure of Zika virus. *Science*.

[31] Limon-Flores A. Y., Perez-Tapia M., Estrada-Garcia I. (2005). Dengue virus inoculation to human skin explants: an effective approach to assess in situ the early infection and the effects on cutaneous dendritic cells. *International Journal of Experimental Pathology*.

[32] Thézé J., Li T., du Plessis L. (2018). Genomic epidemiology reconstructs the introduction and spread of Zika virus in Central America and Mexico. *Cell Host & Microbe*.

[33] Crill W. D., Roehrig J. T. (2001). Monoclonal antibodies that bind to domain III of dengue virus E glycoprotein are the most efficient blockers of virus adsorption to Vero cells. *Journal of Virology*.

[34] Montes-Gómez A. E., Vivanco-Cid H., Bustos-Arriaga J. (2017). Construct and expression of recombinant domains I/II of dengue virus- 2 and its efficacy to evaluate immune response in endemic area: possible use in prognosis. *Acta Tropica*.

[35] Dejnirattisai W., Wongwiwat W., Supasa S. (2015). A new class of highly potent, broadly neutralizing antibodies isolated from viremic patients infected with dengue virus. *Nature Immunology*.

[36] Abbink P., Larocca R. A., Dejnirattisai W. (2018). Therapeutic and protective efficacy of a dengue antibody against Zika infection in rhesus monkeys. *Nature Medicine*.

[37] Jimenez Corona M. E., de la Garza Barroso A. L., Rodriguez Martínez J. C. (2016). Clinical and epidemiological characterization of laboratory-confirmed authoctonous cases of Zika virus disease in Mexico. *PLoS Currents*.

[38] Halstead S. B., Crowe J., Boraschi D., Rappuoli R. Dengue antibody-dependent enhancement: knowns and unknowns. *Antibodies for Infectious Diseases*.

[39] Kliks S. C., Nimmanitya S., Nisalak A., Burke D. S. (1988). Evidence that maternal dengue antibodies are important in the development of dengue hemorrhagic fever in infants. *The American Journal of Tropical Medicine and Hygiene*.

[40] Guzman M. G., Vazquez S. (2010). The complexity of antibody-dependent enhancement of dengue virus infection. *Viruses*.

[41] Montoya M., Collins M., Dejnirattisai W. (2018). Longitudinal analysis of antibody cross-neutralization following Zika virus and dengue virus infection in Asia and the Americas. *The Journal of Infectious Diseases*.

[42] Wahala W. M. P. B., de Silva A. M. (2011). The human antibody response to dengue virus infection. *Viruses*.

[43] Valiant W. G., Lalani T., Yun H. C., Kunz A., Burgess T. H., Mattapallil J. J. (2018). Human serum with high neutralizing antibody titers against both Zika and dengue virus shows delayed in vitro antibody-dependent enhancement of dengue virus infection. *Open Forum Infectious Diseases*.

[44] Pantoja P., Pérez-Guzmán E. X., Rodríguez I. V. (2017). Zika virus pathogenesis in rhesus macaques is unaffected by pre-existing immunity to dengue virus. *Nature Communications*.

[45] Katzelnick L. C., Montoya M., Gresh L., Balmaseda A., Harris E. (2016). Neutralizing antibody titers against dengue virus correlate with protection from symptomatic infection in a longitudinal cohort. *Proceedings of the National Academy of Sciences of the United States of America*.

[46] Deng Y. Q., Dai J. X., Ji G. H. (2011). A broadly flavivirus cross-neutralizing monoclonal antibody that recognizes a novel epitope within the fusion loop of E protein. *PLoS One*.

