# Supplementary material for: Cross-Reaction, Enhancement, and Neutralization Activity of Dengue Virus Antibodies against Zika Virus: A Study in the Mexican Population
Source: J Immunol Res. 2019 Sep 2;2019:7239347. doi: 10.1155/2019/7239347 (PMC6745170; doi:10.1155/2019/7239347)
Supplement: Supplementary Materials — Supplementary Fig. 1: antibody-dependent enhancement of ZIKV by DF and DHF sera. Supplementary Fig. 2: neutralization of ZIKV by DENV sera at different dilutions of DHF sera. [file 7239347.f1.pdf]

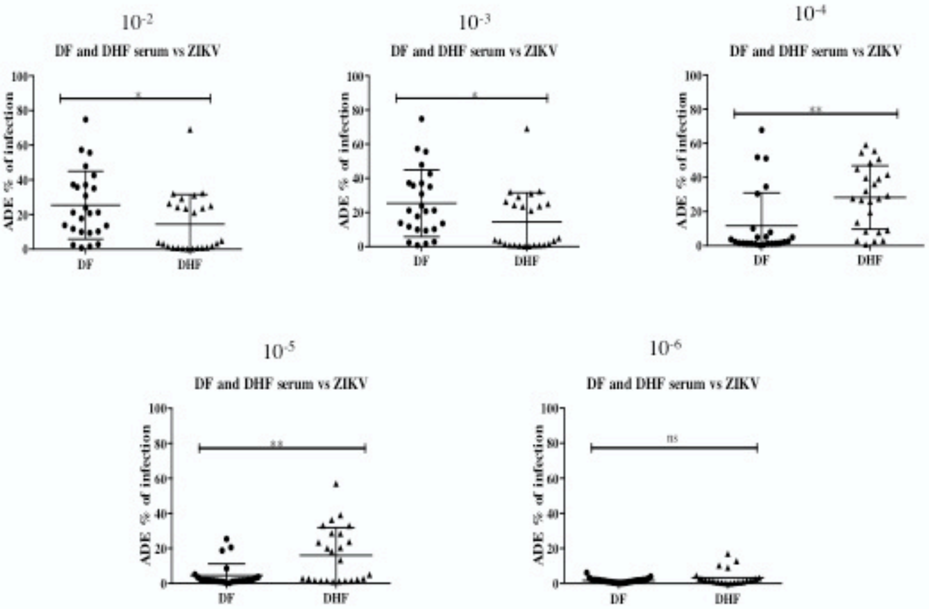

Supplementary Fig 1. Antibody-dependent enhancement of ZIKV by DF and DHF sera. Comparison of both groups DF and DHF. Serially diluted sera (10^{-2}-10^{-6}). were mixed and incubated for 1 h with 1.25 × 10^5 PFU of ZIKV and used to infect 5 × 10^4 K562 cells for 24 h. Infection was analyzed by flow cytometry.. As a positive control, 4G2 serum was used for ADE Bars represent the mean ± SD. *P < 0.05, **P < 0.01, ***P < 0.001.

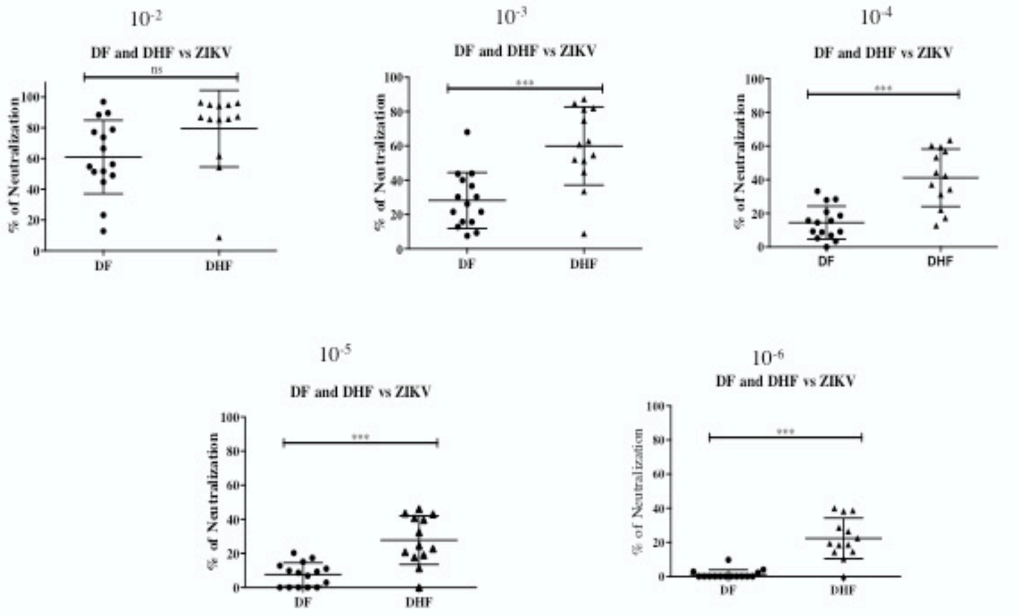

Supplementary Fig 2. Neutralization of ZIKV by DENV sera at different dilutions of DF and DHF sera. Serially diluted sera from (10^{-2}-10^{-6}). DF and DHF patients were mixed with 7.5×10^5 PFU of ZIKV and incubated for 1 h; these mixture were used to infect 2.5×10^5 Vero cells for 24 h, and infection was evaluated by flow cytometry. Bars represent the mean ± SD. *P < 0.05, **P < 0.01, ***P < 0.001.
